# The impact of specific health warning messages on Chinese young people’s perception of smoking risks and quitting intentions

**DOI:** 10.18332/tid/194169

**Published:** 2024-10-23

**Authors:** Zhuo Zhang, Junjie Peng, Gang Wang, Liyun Wu, Kecheng Du

**Affiliations:** 1 School of Journalism and Communication, Wuhan University, Wuhan, China; 2 Faculty of Electrical Engineering and Computer Science, Ningbo University, Ningbo, China; 3 Ethelyn R. Strong School of Social Work, Norfolk State University, Norfolk, United States

**Keywords:** cigarette package warning labels, risk perception, smoking cessation, Chinese youth

## Abstract

**INTRODUCTION:**

Although a substantial body of research has analyzed the effectiveness of cigarette package warning labels in tobacco control, the very general health warnings messages (HWMs) on cigarette packaging in China have shown limited effectiveness in deterring youth from smoking. Therefore, this study investigates the impact of specific and more detailed warning text messages on Chinese young people’s risk perception of smoking and their intention to quit.

**METHODS:**

We employed a randomized survey experiment to examine the impact of specific text-based warning labels on Chinese young people’s risk perception of smoking and intention to quit. The total effective sample size was 1064 participants. The subjects were divided into three groups: the first group served as the control group, which was shown the existing cigarette package warning labels; the second group was shown cigarette package warning labels related to cardiovascular, digestive, and respiratory diseases; and the third group was shown cigarette package warning labels related to sexual dysfunction.

**RESULTS:**

The respiratory disease-related warnings significantly increased young people’s awareness of smoking-related respiratory risks (p<0.01). The impact of warning labels for the three common diseases on enhancing young people’s overall risk perception of smoking (p<0.05) and their intention to quit exhibited only weak statistical significance (p<0.05). In contrast, warning labels related to sexual dysfunction significantly increased young people’s risk perception of smoking (p<0.001) and their intention to quit (p<0.001), with a much higher level of statistical significance compared to those related to the other three common diseases.

**CONCLUSIONS:**

Detailed descriptions of the risks associated with all four diseases were positively correlated with awareness of smoking-related harm and the intention to quit. However, warnings related to sexual dysfunction had a greater level of statistical significance compared to those related to the other three common diseases. This stronger significance may be attributed to young people’s heightened concern about sexual dysfunction.

## INTRODUCTION

Smoking is one of the major factors endangering human health today. In 2019, there were approximately 1.14 billion smokers globally, and smoking caused about 7 million deaths worldwide each year^1,2^. China is the largest producer of tobacco and cigarettes in the world^3,4^, and it is also one of the countries with the highest smoking rates^4,5^. In China, many smokers develop a daily smoking habit around the age of 20 years^6^, making it a significant public health issue, particularly for the younger generation. Despite facing severe health challenges, China lags behind most other countries in implementing strong and effective tobacco control policies^4,7^. For young people, forming a long-term smoking habit might stem from just one attempt^8^. Controlling smoking, especially among the younger population, remains a daunting task in China.

Reviewing existing research in the field of cigarette risk perception and smoking cessation intentions, the study of Weinstein^9^ found that smokers tend to underestimate the harm that smoking poses to themselves, and previous risk-oriented approaches have failed to make people fully aware of their vulnerability to health risks. The Murphy-Hoefer et al.^10^ survey revealed that young smokers do not fully recognize the risks of smoking and that it is crucial to make young smokers aware that every cigarette they smoke is detrimental to their health. Kowitt et al.^11^ found that the more smokers are aware of smoking-related diseases, the more likely they are to attempt to quit. Song et al.^12^ assessed adolescents’ perceptions of the risks of secondhand smoke, finding that if adolescents perceive high personal health risks from secondhand smoke, their likelihood of starting to smoke decreases significantly. Friestad et al.^13^ showed that adolescents’ self-attribution regarding smoking is closely linked to their intentions to quit, with personal health concerns serving as a significant motivation for quitting smoking. The decision to avoid smoking and the attempts to quit are largely driven by an increased perception of smoking-related harms and heightened awareness of personal health. Health warning messages (HWMs) can strengthen young people’s perceptions of smoking risks and concerns about personal health, thereby preventing them from smoking or enhancing their intentions to quit.

In the field of tobacco control, Brodar et al.^14^ found that even if adolescents themselves do not smoke, the use of cigarette packs with warning labels by parents can serve as a warning to adolescents, reinforcing their negative perceptions of smoking and deterring them from trying it. Sychareun et al.^15^ mentioned that the current text-only warning labels in Laos are considered insufficiently informative, providing less information than pictorial warnings and that the format of text labels needs to be improved to make them more specific and persuasive. Vardavas et al.^16^ found that text-only warning labels had a certain degree of effectiveness in increasing awareness of the harms of smoking. However, due to the limitations of text-only labels in conveying content, pictorial warning labels may be more effective in providing warnings. These studies suggest that whether among smokers or non-smokers, warning information on cigarette packaging can serve as an effective deterrent, making it one of the crucial measures in tobacco control. The lower warning effectiveness of text-only labels compared to pictorial ones is largely due to the insufficient amount of information conveyed by text, highlighting the need further to optimize the informational content and persuasiveness of text warnings.

A large body of research, including the aforementioned studies, has demonstrated that pictorial warnings on cigarette packaging are more effective in providing warnings compared to text-only labels^17-22^. Currently, China mandates only text warnings on cigarette product packaging. Although China implemented new cigarette warning label regulations in 2008, increasing the proportion of warning messages on cigarette packaging, both the old and new text-only warnings have failed to achieve highly effective warning outcomes^17,18,20^. However, it has been proven that well-designed warning labels can effectively enhance awareness of the dangers of smoking, refuting claims that better warning labels are ineffective in raising awareness of smoking-related harms^23^. The Chinese government may adopt measures to mandate more prominent pictorial health warnings on cigarette packaging in the future. Under China’s current cigarette packaging regulations, there should also be attempts to further optimize text-only warning labels by increasing their informational content, thereby enhancing young people’s risk perception of smoking and their intention to quit. Nevertheless, research on optimizing text-only warning labels is limited, providing little guidance for China.

In light of the conflicts between previous research and the actual situation in China, this study aims to address the following questions through a survey experiment. How do specific text-based cigarette health warnings, particularly more detailed warning messages, affect Chinese young people’s risk perception of smoking and their intention to quit? Compared to the more commonly known warnings on the harm that smoking causes to the respiratory, digestive, and cardiovascular systems, are warnings about the harm that smoking does to the reproductive system more effective in enhancing Chinese young people’s risk perception of smoking and their intention to quit?

## METHODS

### Research design

This study employed a survey experiment method which combines the strengths of both surveys and experiments, thereby allowing for a larger sample size. In the experiment, we selected three popular price categories of cigarettes in China – high, medium, and low. In current public health campaigns in China, such as those found on Baidu, the largest search engine in China, the most commonly found information about the health risks of smoking pertains to respiratory diseases, digestive diseases, and cardiovascular diseases. However, information on the reproductive system’s risks from smoking is relatively rare. We hypothesize that compared to older adults, information about the harm smoking causes to the reproductive system may be more effective in deterring young people from smoking.

**Figure 1:**
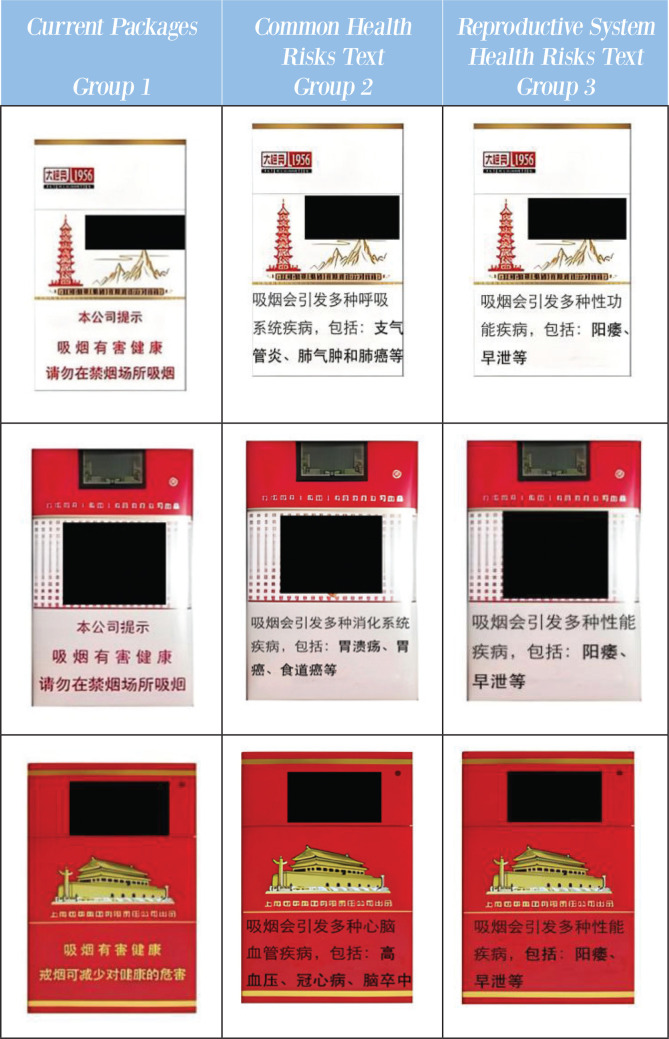
Warning packages and prices applied in survey experiment among Chinese youth smokers aged 18–35 years, China, 2024 (N=1064)

Based on these considerations, the study divided the participants into three groups, each receiving a questionnaire containing different cigarette packaging images (Figure 1). The first group served as the control group. It was shown that various commonly available cigarette packages from the market were available, establishing a baseline for evaluating the potential impact of packaging on smokers’ risk perception. The warning message on the packaging was: ‘Our company reminds you: Smoking is harmful to your health. Please do not smoke in public places’.

The second group, the ‘common health risks text’ group, had the text message on the cigarette packaging replaced with detailed textual descriptions of three common diseases caused by smoking: respiratory, digestive, and cardiovascular. The warning messages on the packaging were: ‘Smoking can cause a variety of respiratory diseases, including bronchitis, emphysema, and lung cancer, etc.’, ‘Smoking can cause a variety of digestive system diseases, including gastric ulcers, gastric cancer, esophageal cancer, etc.’, and ‘Smoking can cause a variety of cardiovascular and cerebrovascular diseases, including hypertension, coronary heart disease, stroke’. The three warning messages were placed on cigarette packs at three different price points, allowing the respondents in this group to see all three common disease warnings.

The third group focused on the reproductive system health risks. The warning message on the packaging was: ‘Smoking can cause a variety of sexual dysfunctions, including impotence, premature ejaculation, etc.’.

During the study, we found that although smoking harms the reproductive system, most smokers lack awareness of this, and related information is less frequently encountered in online media. However, we hypothesized that describing the harm to the reproductive system might be more effective in raising young people’s awareness of the dangers of smoking, especially among those without children. To test this, this group’s warning labels on cigarette packaging were replaced with detailed descriptions of reproductive system diseases.

### Sample participants and data collection

The study primarily targeted Chinese youth smokers aged 18–35 years for sample collection. Participants were recruited through a snowball sampling method and completed relevant questionnaires. The field researchers were all undergraduate students who voluntarily signed up, from universities in Wuhan, a city in central China. The researchers randomly selected participants from this pool, based on their student identification numbers, to serve as research assistants responsible for administering the questionnaires. In May 2024, we initially randomly selected 80 undergraduates from a large research university in China, who then collected data through their social networks using a snowball approach. Before data collection, these students were randomly divided into three groups, and three questionnaires were distributed to their networks. The control group was assigned 26 investigators, while the other two were each assigned 27 investigators. The data collection period lasted for one month, and the total sample size was 1064 participants. The first group (control group), which maintained the current cigarette packaging warning, consisted of 343 participants. In the second group (common health risks text group), the existing warning labels on cigarette packaging were replaced with detailed descriptions of three common smoking-related diseases, each corresponding to a different price tier of cigarette packaging. This group consisted of 342 participants. In the third group (reproductive system health risks text group), the existing warning labels were replaced with detailed descriptions of diseases related to the reproductive system. This group included a total of 379 participants.

### Measurement

The dependent variables in this study include participants’ perceptions of the harms of smoking and their intention to quit smoking. After answering some basic information questions, participants in the three experimental groups were shown the corresponding cigarette packaging images attached to the questionnaires. They were then asked about their perception of the harms of smoking and their intention to quit after being exposed to the experimental conditions.

The question measuring the perceived harm of smoking, in general, was: ‘To what extent do you agree that cigarette package warnings make you think of the severity of harm from smoking?’, using a five-point Likert scale (1=strongly disagree, 2=somewhat disagree, 3=neutral, 4=somewhat agree, and 5=strongly agree). Additionally, we measured participants’ intention to quit smoking with the question: ‘To what extent do you agree that cigarette package warnings make you think of quitting smoking?’, using the same a five-point Likert scale. In addition, we separately examined the respondents’ perceptions of health risks related to respiratory diseases, digestive diseases, cardiovascular diseases, and reproductive system diseases. The questions posed were: ‘To what extent do you think tobacco will harm your respiratory system?’, ‘To what extent do you think tobacco will harm your digestive system?’, ‘To what extent do you think tobacco will harm your cardiovascular system?’, and ‘To what extent do you think tobacco will harm your sexual function?’. These questions were measured using a four-point Likert scale (1=very little, 2=somewhat little, 3=somewhat much, and 4=very much). The measurement of these variables refers to questionnaires used in several established studies^20,24,25^.

The survey also collected demographic information about the participants, including age, gender, education level, annual income, marital status, and whether they have children. The age variable was measured in years based on the reported birthdate; gender was a binary variable (1=male, 0=female); education level was measured on a seven-point scale (1=Primary school, 2=Middle school, 3=High school, 4=Associate degree, 5=Bachelor’s degree, 6=Master’s degree, 7=Doctoral degree); annual income was measured in units of wan (1 wan=10000 RMB;1 RMB about US$0.15). This question was designed as an open-ended item, allowing respondents to self-report their exact annual income; marital status was a binary variable (0=single, 1=married/cohabiting/divorced/widowed); and parental status was also a binary variable (0=no children, 1=has children).

We also used nicotine dependence as a control variable for statistical inference analysis, including the average daily usage of cigarettes and the average time to smoke the first cigarette after waking up in the morning. The average time (minutes) to smoke the first cigarette after waking up in the morning was measured using a four-point item (1= ≤ 5; 2 = 6–30; 3= 31–60; 4= ≥61). Average daily cigarette consumption was also measured using a four-point indicator (1= ≤10; 2= 11–20; 3= 21–30, and 4= ≥31).

### Statistical analysis

All statistical analyses were conducted using Stata 15.1 software. The descriptive statistics section reports the means and standard deviations. Multivariable relationships are estimated using multivariable linear regression models, with 95% confidence intervals. All regression models include demographic characteristics and nicotine dependence as control variables. The significance levels are categorized into p<0.001, p<0.01, p<0.05. Smaller p-values indicate strong evidence to reject the null hypothesis, thereby supporting the alternative hypothesis.

## RESULTS

### Descriptive analysis

Table 1 presents each subgroup’s means and the overall mean of the dependent variables. The results show that the mean perception of harm to the cardiovascular system was 3.39 (SD=1.41); the mean perception of harm to the digestive system was 3.55 (SD=1.21); the mean perception of harm to the respiratory system was 4.10 (SD=1.07); and the mean perception of harm to the reproductive system was 3.37 (SD=1.37). The mean perception of personal smoking harm was 3.87 (SD=1.19); and the mean intention to quit smoking was 3.73 (SD=1.25).

**Table 1:** Descriptive statistics* of Chinese youth smokers aged 18–35 years for a survey experiment of the impact of specific HWMs on perceived harm of smoking to different systems, China, 2024 (N=1064)

*Characteristics*	*Group 1* *Mean (SD)*	*Group 2* *Mean (SD)*	*Group 3* *Mean (SD)*	*All* *Mean (SD)*
Harmful to the cardiovascular system	3.28 (1.45)	3.54 (1.28)	3.37 (1.49)	3.39 (1.41)
Harmful to the digestive system	3.44 (1.20)	3.66 (1.12)	3.50 (1.30)	3.55 (1.21)
Harmful to the respiratory system	4.04 (1.11)	4.25 (0.87)	4.03 (1.17)	4.10 (1.07)
Harmful to the reproductive system	3.19 (1.43)	3.16 (1.31)	3.72 (1.29)	3.37 (1.37)
Perceived harm of personal smoking	3.62 (1.34)	3.85 (1.18)	4.10 (0.98)	3.87 (1.19)
Willingness to quit smoking	3.46 (1.37)	3.69 (1.23)	4.00 (1.10)	3.73 (1.25)
Age (years)	26.72 (5.07)	26.20 (4.51)	26.80 (4.60)	26.58 (4.73)
Gender	0.91 (0.29)	0.87 (0.34)	0.92 (0.27)	0.90 (0.30)
Education level	4.12 (1.19)	4.45 (1.11)	4.10 (1.29)	4.22 (1.21)
Annual income (wan RMB)	9.55 (12.43)	9.55 (11.12)	8.76 (14.69)	9.27 (12.89)
Marital status	0.69 (0.47)	0.53 (0.50)	0.59 (0.49)	0.60 (0.49)
Parental status	0.67 (0.47)	0.53 (0.50)	0.58 (0.50)	0.59 (0.49)
Cigarette daily consumption	1.76 (0.87)	1.65 (0.82)	1.69 (0.81)	1.70 (0.83)
Average waiting time from wakening to smoking the first cigarette	2.34 (1.14)	2.11 (1.08)	2.30 (1.10)	2.25 (1.11)
Total	343	342	379	1064

* For scale scores see the Measurement section. Income: 1 wan=10000 RMB;1 RMB about US$0.15. HWM: health warning message.

The data show that 90.1% of the respondents are male; the average education level is between college and undergraduate; the average annual income is 9.27 (12.89) wan RMB; 60% of the respondents were not single; 59.2% of the respondents had children. The scale score for the average number of cigarettes smoked per day was 1.7 (SD=0.83) (about 11 cigarettes/day), and the mean time to the first cigarette after waking up in the morning was between 30 and 60 minutes.

### Multivariable regression analysis

Tables 2–5 display the estimated results of the multivariable regression analyses. During the analysis, cases with missing responses to specific questions were automatically excluded from the sample. Table 2 and Model 1 in Table 4, and Model 3 in Table 5 are based on subsamples from the control group (Group 1) and the common health risks text group (Group 2). After excluding samples with missing values, the total sample size was n=678. Table 3, Model 2 in Table 4, and Model 4 in Table 5 are based on subsamples from the control group and the reproductive health risks text group (Group 3). After excluding samples with missing values, the total sample size was n=712.

**Table 2:** A survey experiment of the impact of specific HWMs on perceived harm of smoking to different systems among Chinese youth smokers aged 18–35 years, Group 1 vs Group 2, China, 2024 (N=678)

	*Cardiovascular system*		*Digestive system*		*Respiratory system*		*Reproductive system*	
	*β (95% CI)*		*β (95% CI)*		*β (95% CI)*		*β (95% CI)*	
Specific messages	0.15 (-0.05–0.36)	0.15	0.16 (-0.01–0.34)	0.07	0.22** (0.07–0.37)	0.01	0.01 (-0.20– 0.22)	0.91
**Control variable**								
Age (years)	-0.00 (-0.03–0.02)	0.72	-0.02 (-0.04–0.01)	0.06	0.00 (-0.01– 0.02)	0.82	-0.00 (-0.03–0.02)	0.76
Gender	0.40 (0.07–0.74)	0.02	0.33* (0.04–0.62)	0.02	-0.18 -0.43–0.08)	0.17	0.21 (-0.14–0.55)	0.24
Education level	0.18*** (0.08–0.27)	0.001	0.03 (-0.05–0.12)	0.44	0.00 (-0.07–0.07)	0.99	-0.07 (-0.17–0.03)	0.19
Income	0.00 (-0.01–0.01)	0.97	0.01* (0.00–0.02)	0.01	-0.00 (-0.00–0.10)	0.46	0.00 (-0.01–0.01)	0.48
Marital status	0.21 (-0.19–0.61)	0.30	0.11 (-0.23–0.46)	0.51	-0.11 (-0.41–0.19)	0.46	-0.30 (-0.71–0.12)	0.16
Children	-0.55** (-0.95 – -0.14)	0.01	-0.51** (-0.86 – -0.17)	0.004	0.07 (-0.23–0.38)	0.63	0.20 (-0.22–0.61)	0.36
Cigarette daily consumption	-0.03 (-0.17–0.12)	0.73	-0.01 (-0.13–0.11)	0.83	0.16** (0.05–0.27)	0.003	0.06 (-0.08–0.21)	0.41
Average waiting time from wakening to smoking the first cigarette	-0.02 (-0.13–0.08)	0.67	0.10* (0.01–0.19)	0.03	0.04 (-0.04–0.12)	0.29	0.14* (0.03–0.25)	0.01
Adj R^2^	0.05		0.04		0.03		0.015	
Total	678		678		678		678	

HWM: health warning message.

*** p<0.001.

** p<0.01.

* p<0.05.

**Table 3:** A survey experiment of the impact of specific HWMs on perceived harm of smoking to different systems among Chinese youth smokers aged 18–35 years, Group 1 vs Group 3, China, 2024 (N=712)

	*Cardiovascular system*		*Digestive system*		*Respiratory system*		*Reproductive system*	
	*β (95% CI)*		*β (95% CI)*		*β (95% CI)*		*β (95% CI)*	
Specific messages	-0.01 (-0.11–0.10)	0.88	0.01 (-0.08–0.10)	0.81	-0.01 (-0.09–0.08)	0.91	0.27*** (0.17–0.37)	0.001
**Control variable**								
Age (years)	-0.01 (-0.03 – -0.02)	0.59	-0.02 (-0.04–0.00)	0.09	-0.00 (-0.02–0.02)	0.89	-0.00 (-0.02–0.02)	0.92
Gender	0.36 (-0.02–0.75)	0.06	0.37* (0.03–0.70)	0.04	0.16 (-0.16–0.48)	0.31	-0.15 (-0.53–0.23)	0.44
Education level	0.15*** (0.06–0.25)	0.001	-0.02 (-0.11–0.06)	0.56	0.00 (-0.07–0.08)	0.94	-0.01 (-0.10–0.82)	0.83
Income	0.01* (0.00–0.02)	0.03	0.01*** (0.01–0.02)	0.001	0.00 (-0.01–0.01)	0.99	0.00 (-0.01–0.01)	0.50
Marital status	-0.16 (-0.56–0.23)	0.42	-0.16 (-0.51–0.19)	0.36	-0.20 (-0.53–0.13)	0.24	-0.17 (-0.56–0.22)	0.38
Children	-0.74*** (-1.13 – -0.34)	0.001	-0.26 (-0.61–0.09)	0.14	0.20 (-0.13–0.52)	0.24	0.14 (-0.25–0.53)	0.48
Cigarette daily consumption	-0.06 (-0.21–0.08)	0.40	0.04 (-0.09–0.17)	0.57	0.04 (-0.08–0.17)	0.48	0.02 (-0.13–0.16)	0.84
Average waiting time from wakening to smoking the first cigarette	0.08 (-0.03–0.18)	0.14	0.12* (0.03–0.21)	0.01	0.08 (-0.01–0.17)	0.07	0.10 (-0.00–0.21)	0.05
Adj R^2^	0.12		0.05		0.00		0.04	
Total	712		712		712		712	

HWM: health warning message.

*** p<0.001.

**p<0.01.

* p<0.05.

**Table 4:** A survey experiment of the impact of specific HWMs on young peoples’ perceived harm of smoking in general among Chinese youth smokers aged 18–35 years, China, 2024 (N=1064)

	*Model 1 (Group 1 vs 2)*		*Model 2 (Group 1 vs 3)*	
	*β (95% CI)*		*β (95% CI)*	
Specific messages	0.22* (0.03–0.42)	0.03	0.22*** (0.14–0.31)	0.001
**Control variable**				
Age (years)	-0.01 (-0.03–0.01)	0.31	-0.01 (-0.03–0.01)	0.48
Gender	0.12 (-0.20–0.44)	0.46	0.39* (0.07–0.71)	0.02
Education level	0.00 (-0.09–0.09)	1.00	-0.00 (-0.08–0.08)	0.96
Income	0.01 (-0.00–0.01)	0.30	0.00 (-0.00–0.01)	0.46
Marital status	0.20 (-0.18–0.58)	0.31	0.05 (-0.29–0.38)	0.79
Children	-0.23 (-0.62–0.15)	0.24	-0.15 (-0.49–0.18)	0.37
Cigarette daily consumption	0.021 (-0.113–0.156)	0.76	-0.05 (-0.17–0.08)	0.46
Average waiting time from wakening to smoking the first cigarette	0.02 (-0.08–0.12)	0.64	0.04 (-0.04–0.13)	0.33
Adj R^2^	0.00		0.04	
Total	678		712	

HWM: health warning message.

*** p<0.001.

**p<0.01.

* p<0.05.

**Table 5:** A survey experiment of the impact of specific HWMs on Chinese youth’s willingness to quit smoking among Chinese youth smokers aged 18–35 years, China, 2024 (N=1064)

	*Model 3 (Group 1 vs 2)*		*Model 4 (Group 1 vs 3)*	
	*β (95% CI)*		*β (95% CI)*	
Specific messages	0.22* (0.02–0.42)	0.03	0.27*** (0.18–0.36)	0.001
**Control variable**				
Age (years)	-0.00 (-0.03–0.02)	0.74	0.00 (-0.02–0.02)	0.79
Gender	0.32 (-0.01–0.65)	0.06	0.29 (-0.06–0.63)	0.10
Education level	0.02 (-0.08–0.12)	0.72	0.06 (-0.03–0.14)	0.19
Income	0.01 (-0.00–0.02)	0.07	0.00 (-0.00–0.01)	0.36
Marital status	0.11 (-0.28–0.50)	0.59	0.02 (-0.34–0.37)	0.92
Children	-0.19 (-0.59–0.20)	0.34	0.08 (-0.27–0.44)	0.65
Cigarette daily consumption	-0.01 (-0.15–0.13)	0.89	-0.06 (-0.19–0.08)	0.40
Average waiting time from wakening to smoking the first cigarette	-0.00 (-0.11–0.10)	0.95	-0.01 (-0.10–0.09)	0.90
Adj R^2^	0.01		0.04	
Total	678		712	

HWM: health warning message.

*** p<0.001.

**p<0.01.

* p<0.05.

Table 2 presents the estimated results of participants’ perception of smoking-related harms to different systems. After the common health risks text intervention, participants’ perception of harm to the respiratory system significantly increased (respiratory system, β=0.22; 95% CI: 0.07–0.37, p<0.01). However, the perceived harm to the digestive and cardiovascular systems did not increase significantly. Regarding control variables, the results in Table 2 indicate that male respondents(digestive system, β=0.33; 95% CI: 0.04–0.62), those with higher education (cardiovascular system, β=0.18; 95% CI: 0.08–0.27), individuals with higher income (digestive system, β=0.01; 95% CI: 0.00–0.02), those without a child (cardiovascular system, β= -0.55; 95% CI: -0.95 – -0.14; digestive system, β= -0.51, 95% CI: -0.86 – -0.17), and who have strong nicotine dependence as indicated by the number of cigarettes per day (respiratory system, β=0.16; 95% CI: 0.05–0.27) and longer wait time to first cigarette (digestive system, β=0.10; 95% CI: 0.01–0.19; reproductive system, β=0.14, 95% CI: 0.03– 0.25), exhibit a higher perception of harm to different systems.

Table 3 presents the estimated impact on the perception of smoking-related harms to different systems. The results show that after adding the reproductive health risks text, participants’ perception of harm to the reproductive system significantly increased (β=0.27, 95% CI: 0.17–0.37, p<0.001). Regarding control variables, the results in Table 3 show that male respondents (digestive system, β=0.37; 95% CI: 0.03–0.70), those with high education level (cardiovascular system, β=0.15; 95% CI: 0.06–0.25), individuals with high income (cardiovascular system, β=0.01; 95% CI: 0.00–0.02; digestive system, β=0.01; 95% CI: 0.01–0.02), those without child (cardiovascular system, β= -0.74; 95% CI: -1.13 – -0.34), and who have low nicotine dependence based on time to first cigarette (digestive system, β=0.12; 95% CI: 0.03–0.21), exhibit a higher perception of harm to different systems.

Table 4 presents the estimated impact on the overall perception of smoking-related harms. Model 1 shows that the effect of common health risks text on participants’ perception of smoking-related harms demonstrates weak significance (Model 1, β=0.22; 95% CI: 0.03–0.42, p<0.05). In contrast, Model 2 indicates that after the addition of reproductive health risks text, smokers’ perception of smoking-related harms significantly increased, showing strong significance (Model 2, β=0.22; 95% CI: 0.14–0.31, p<0.001); and male participants (Model 2, β=0.39; 95% CI: 0.07–0.71) may exhibit higher reproductive system harm perceptions.

Table 5 presents the estimated impact on respondents’ intention to quit smoking. Model 3 shows that the common health risks text demonstrates relatively weak significance in influencing respondents’ intention to quit smoking (Model 3, β=95% CI: 0.02–0.42, p<0.05). However, Model 4 indicates that after the addition of reproductive health risks text, respondents’ intention to quit smoking significantly increased, with stronger significance (Model 4, β=0.27; 95% CI: 0.18–0.36, p<0.001).

## DISCUSSION

We used a randomized survey experiment to examine the impact of two groups of specific text-based cigarette health warning messages on Chinese young people’s risk perception of smoking and their intention to quit. Multivariable regression results show that health warning messages related to cardiovascular, digestive, and respiratory diseases only exhibited weak statistical significance in their correlation with general awareness of smoking-related harm and the willingness to quit smoking. These three types of health warnings may indeed have increased respondents’ risk perception of smoking and their desire to quit, but this relationship is not statistically robust. In contrast, cigarette warning messages related to male sexual dysfunction had a more statistically significant impact on young people’s risk perception of smoking and their willingness to quit, suggesting that such warnings effectively heightened young people’s awareness of the risks of smoking and their intention to quit.

Compared to the current vague and generalized warnings, the specific and more detailed cigarette health warning messages used in this study provided detailed information on specific diseases related to smoking, thereby increasing the informational content of the warnings. This allowed participants to gain a clearer understanding of the harms associated with smoking. The results of the survey indicate that adding more detailed and explicit health risk information increased Chinese young people’s perception of the harms of smoking, regardless of the specific diseases involved, making the warning messages more effective. These findings provide further experimental evidence supporting previous studies conducted in countries such as the United Kingdom^26^ and Spain^27^.

Building on this findings, the study also discovered that the warning effects of different health risk messages varied, which may be related to the age of the participants. Cardiovascular and digestive system diseases typically develop with age, making them seem distant and less likely to affect young people, diminishing the impact of these warnings on younger individuals. In contrast, respiratory system diseases may be more relevant to young people, leading to an increased awareness of the risks associated with respiratory health. Sexual function is closely related to young people’s future relationships and family-building, making sexual dysfunction a more salient concern. As a result, health warning messages related to sexual function are more likely to capture young people’s attention, significantly increasing their risk perception of smoking and enhancing their intention to quit. This study not only confirms the conclusion of Friestad and Rise^13^ that concern about personal health issues is an important motivation for teenagers to quit smoking, but it further shows that raising awareness of closely related personal health issues, such as diseases associated with sexual function, can be even more effective in helping them quit smoking.

Additionally, the reproductive disease addressed in this study is specific to males, suggesting a difference in the effectiveness of the warning across genders. In this study, female respondents accounted for only about 10%, with male respondents making up a significant proportion. This gender distribution is consistent with smoking prevalence worldwide, particularly in China, where male smoking rates are significantly higher than female rates^28-30^. Therefore, the findings of this study still have considerable practical relevance.

In China, it is essential not only to strengthen young people’s awareness of the harms of smoking but also to promote tobacco-related health knowledge across all age groups. Surveys of smoking risk perception among Chinese smokers and non-smokers^31,32^ indicate that people of all ages have a relatively poor grasp of tobacco-related knowledge, hindering the promotion of smoking cessation measures. Future research could focus on the impact of specific cigarette warning content on the risk perception of smoking and the intention to quit in other age groups, potentially leading to further discoveries based on this study.

### Limitations

This study has several limitations. First, the survey was conducted among a young Chinese population, and the results may vary across different age groups, limiting the generalizability of the findings to the overall smoking population. Second, the warning effectiveness of reproductive health information is influenced by the gender of the smokers, which means that the findings may not apply to regions where the gender distribution of smokers is more balanced or where female smokers predominate. Third, this study only examined the impact of text-only labels on young people’s risk perception of smoking and their intention to quit in China. Combining these labels with related pictorial warnings might yield different results. Fourth, the study employed a snowball sampling method, which may have affected the sample’s representativeness. Fifth, the study relied on self-reported survey responses, which could be subject to measurement bias due to social desirability effects. Sixth, whether this study’s conclusions apply to other cultural contexts remains to be verified.

## CONCLUSIONS

This study employs a randomized survey experiment to examine the impact of two groups of specific text-based cigarette package warning labels on Chinese young people’s risk perception of smoking and their intention to quit. The results indicate that detailed descriptions of the harms of four diseases are positively correlated with young people’s perception of smoking-related harm and their intention to quit, reaching the threshold for statistical significance. However, warnings related to sexual dysfunction had a far greater level of statistical significance compared to those related to the other three common diseases. This difference may be attributed to young people’s heightened concern about sexual dysfunction.

## Data Availability

The data supporting this research are available from the authors on reasonable request.
